# The Promise and Pursuit of MicroRNAs for Cancer Treatment

**DOI:** 10.3390/biom16081086

**Published:** 2026-07-24

**Authors:** Camaleta Boothe, Arianna Rossi, Jenniffer Kalil, Jean J. Latimer

**Affiliations:** 1 Pharmaceutical Sciences, Nova Southeastern University, Fort Lauderdale, FL 33328, USAar4734@mynsu.nova.edu (A.R.); jkalil@nova.edu (J.K.); 2 NSU AutoNation Institute for Breast Cancer Research and Care, Nova Southeastern University, Fort Lauderdale, FL 33328, USA

**Keywords:** microRNA, cancer therapeutics, microRNA mimics, antagomirs, oncomiRs, tumor suppressor microRNAs, RNA therapeutics, gene regulation, 5-FU-modified microRNA, RNAi

## Abstract

In spite of at least six discrete classes of drugs available for cancer treatment, the quest for more biologic drugs continues. One type of biologic molecule that occurs naturally in the body is microRNA. MicroRNAs regulate post-transcriptional gene expression and can be under expressed in cancer (tumor suppressor microRNAs) or over expressed (oncogenic microRNAs). Strand-specific mimics of microRNAs have been developed and used successfully in vitro, in vivo, and in clinical trials, to control multiple aspects of cancer including metastasis, apoptosis and proliferation. Each microRNA is capable of binding a specific target mRNA or mRNAs, sometimes simultaneously interfering with multiple genes in a single pathway, or binding with a single nodal mRNA. Some microRNAs can facilitate chemotherapy that has stopped working, addressing the issue of drug resistance. Without chemical modification, microRNAs are too vulnerable to have lasting therapeutic value. Chemical modifications to microRNAs have provided nuclease resistance and greater stability and are the basis for microRNA mimics that can be used therapeutically. However, without a vehicle, microRNA mimics do not cross cell membranes. These nanoparticles can cause inflammatory reactions in patients. Additional modifications that enabled microRNA mimics to cross cell membranes include substituting uracil with 5-fluorouracil. Lessons from an siRNA therapeutic called Patisiran offer a roadmap for future success for microRNAs in cancer. This review provides a historical perspective of the continuing evolution of microRNA mimics for cancer treatment.

## 1. Introduction

Cancer continues to be the second most burdensome disease globally with approximately 20 million new cases and 9.7 million deaths annually [1]. Despite advances in cancer screening, diagnosis and treatment, including radiation, chemotherapy, endocrine therapy, immune and targeted therapies, treatment resistance remains the greatest clinical challenge. Consequently the search for more effective, less toxic treatment options continues.

RNAs can regulate gene expression in plants and mammals. It was not until the discovery of the microRNA *let-7* that the fundamental importance of microRNA across most genomes including humans was recognized [2]. While these findings were considered unusual, scientists in the early 2000s found many similar small RNAs in both plants and other animals. The important functional role that microRNAs play has since been revealed to be their role in regulation of gene expression with subsequent applications in many diseases as well as use in drug therapy, disease diagnosis and prognosis [3].

**Figure 1:**
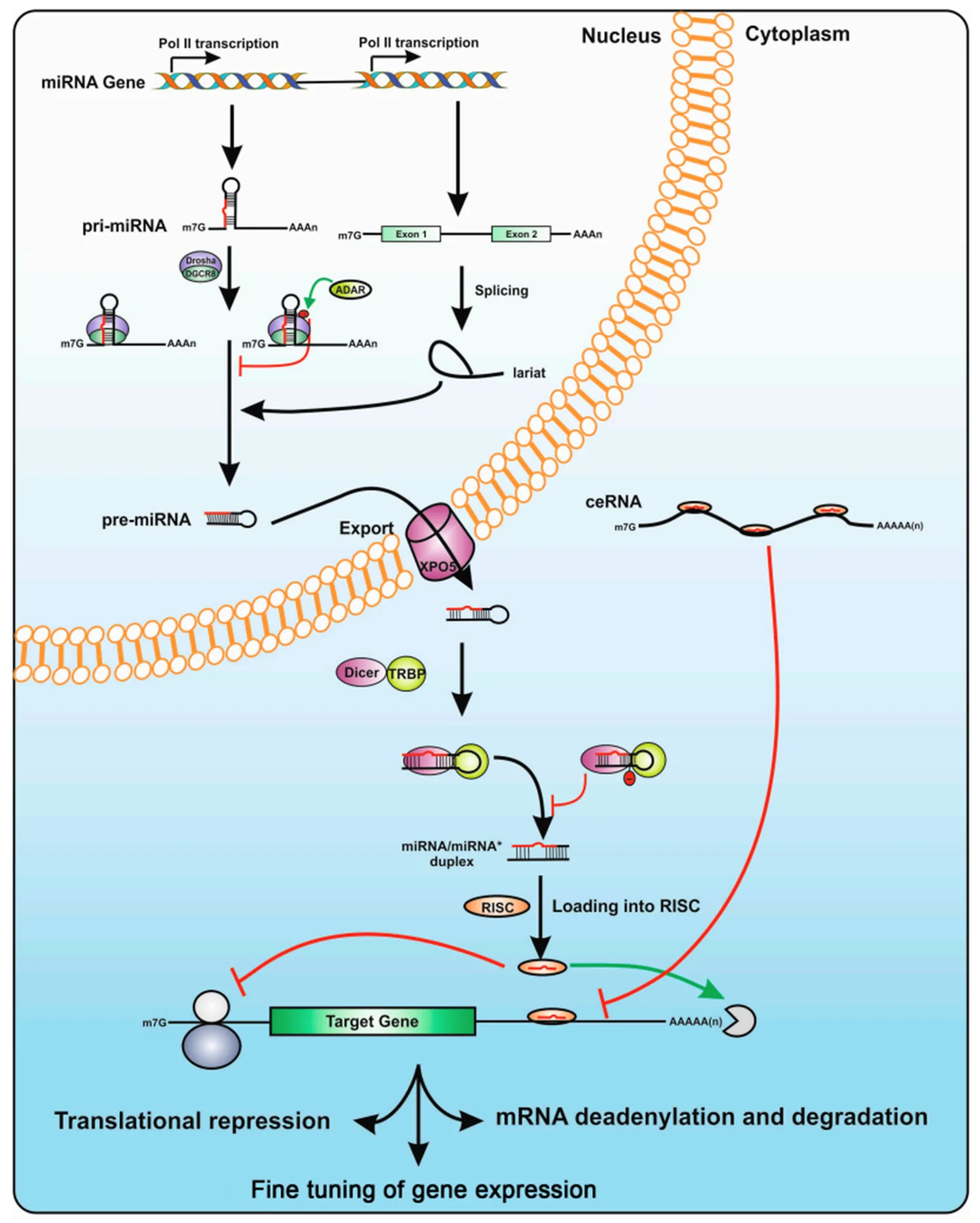
MicroRNA biogenesis pathway. MicroRNAs are transcribed from non-protein coding genes. Primary microRNAs (pri-miRNAs) are processed by the Drosha–DGCR8 complex in the nucleus to generate precursor microRNAs (pre-miRNAs), which are exported to the cytoplasm by Exportin-5 and further processed by Dicer to produce mature microRNAs. Mature microRNAs are incorporated into the RNA-induced silencing complex (RISC), where they regulate target mRNAs through translational repression and/or mRNA degradation. Competing endogenous RNAs (ceRNAs) can further modulate microRNA activity and gene regulation. Reproduced from Gama-Carvalho et al. [7]. The asterisk identifies the passenger strand of the microRNA duplex. Red lines indicate inhibitory interactions, whereas the green arrow indicates a positive regulatory effect.

In humans, microRNAs are transcribed from DNA into RNA molecules through a series of processes (Figure 1). Two essential enzymes, Drosha and Dicer, are responsible for the cleaving of these larger RNAs into smaller pieces, namely, primary, pre- and mature microRNA [4,5]. MicroRNAs typically bind to the 3′ untranslated regions of targeted gene transcripts and subsequently block translation or lead to cleavage of the bound mRNA (Figure 1). These are called suppressor microRNAs. However, research has shown that some microRNAs bind to or interact with the 5′ untranslated region as well as coding regions of targeted messenger RNA. In addition, under specific cellular conditions, microRNAs may promote post-transcriptional gene expression, a mechanism that may contribute to disease development and progression, including cancer [6].

MicroRNAs may bind imperfectly to mRNAs, as they are partially complementary with the ability to bind multiple mRNA targets. Perfect binding of microRNA with mRNA causes argonaut-mediated endonucleolytic cleavage of the mRNA, which prevents translation, and imperfect binding to a seed region triggers the RNA-induced silencing complex (RISC) causing suppression of its translation. MicroRNAs come in two varieties, 5 prime (5p) and 3 prime (3p), depending on which strand actively binds the target mRNA. MicroRNA mimics are usually strand specific.

## 2. MicroRNAs in Cancer

Ongoing studies have found that microRNAs are downregulated in some cancers (tumor suppressors) and upregulated in others (oncogene-like), providing new insights and investigational opportunities for their use as prognostic factors or therapeutics. MicroRNA dysregulation has been reported across a wide range of solid and hematologic malignancies, where microRNAs may function as tumor suppressors or oncogenic microRNAs [8] (Table 1). This dysregulation has been documented in most tumor types including solid tumors (e.g., breast cancer) and hematologic cancer.

Breast cancer is one of the best characterized cancers for microRNAs [91]. Recent studies highlight the roles of breast-cancer-associated microRNAs in diagnosis, prognosis, treatment response, and targeted therapy [92]. Notably, oncogenic microRNAs in breast cancer such as *miR-10b*, *miR-155* and *miR-221* are reported to be frequently overexpressed in breast cancer, while the *miR-200* family, *miR-34a* and *let-7* among others are often downregulated. *MicroRNA-155* is now widely regarded as an upregulated microRNA that is frequently overexpressed in aggressive breast cancers and associated with a poor prognosis [53].

MicroRNA mimics and anti-microRNAs (antagomirs) were created to specifically overcome the hurdles of working with natural microRNAs, allowing for strand specificity and overcoming the lability of RNA. Mimics are synthetic double-stranded RNA molecules designed to imitate the functions of suppressor microRNAs. Mimics are now essential in understanding gene regulation and their use in cancer therapeutics. MRX34, for example, mimics the tumor suppressor *miR-34a* which is downregulated in non-small-cell lung cancer (NSCLC), melanoma and other solid tumors. *MicroRNA-145* mimic is used to block metastasis and has been studied in several cancers including colon, breast and ovarian cancer [46,49,93] (Table 2). The relevance and clinical applications of these synthetic molecules have been demonstrated in their successful use in solid tumor clinical trials (Table 2).

Antagomirs on the other hand can inhibit the activities of respective overexpressed oncomirs, thereby reducing or blocking the translation of specific proteins. An example is shown by anti-miR-10b that targets and blocks the activities of *microRNA-10b*, which is implicated in metastatic breast cancer [17,18].

Several studies have shown that microRNA-129-5p is a tumor suppressor in non-small-cell lung cancer where it is downregulated. Zhang et al. [100] in their investigational studies and use of mimics, demonstrated that increasing or restoring the levels of microRNA-129-5p increased E-cadherin, an epithelial marker, while decreasing vimentin, a mesenchymal marker, and microspherule protein 1 (*MCRS1*). These findings suggest that microRNA-129-5p plays a significant role in reducing metastasis. In addition, Li et al. [34] demonstrated in their in vitro studies that the reduction or loss of microRNA-129-5p contributed to the progression of NSCLC and that increasing its level using mimics significantly reduced proliferation and increased apoptosis pathways. MicroRNA-129-5p also increased the tumor cell sensitivity to the chemotherapeutic agent etoposide causing an apoptotic response by reducing the expression of *YWHAB*, (Tyrosine 3-Monooxygenase/Tryptophan 5-Monooxygenase Activation Protein Beta) a gene that regulates cell proliferation and death [37].

MicroRNA-145-5p also works as a tumor suppressor in hepatocellular carcinoma (HCC). Wang et al. [48] found that microRNA-145-5p levels were low in both HCC tumors and cell lines. MicroRNA-145-5p levels inversely correlated with prognosis and tumor aggressiveness (lower being worse). Restoring microRNA-145-5p slowed cell growth, migration, and invasion by switching off *ARF6*. Reduced microRNA-145-5p was caused by a circular RNA called circPVT1, which competitively sequestered this mature microRNA, and diminished the functional intracellular pool of microRNA-145-5p Li and Ma [74].

MicroRNA levels sometimes depend on competing endogenous RNAs (ceRNAs), a group that includes circular RNAs (circRNAs). CircRNAs are closed loops of RNA with no free ends. CircRNAs, including circPVT1 and hsa_circ_101555, can competitively sequester specific tumor-suppressive microRNAs, reducing their functional intracellular availability and attenuating repression of downstream target mRNAs. These have been shown in hepatic cancers [45,99].

## 3. Clinical Use of MicroRNAs as Therapeutics

MRX34, a *miR-34a* mimic showed promising results in mouse xenograft studies with tumor suppression and eventually entered a human clinical trial (Table 1). *MiR-34a* is an inhibitory microRNA that inhibits cell proliferation by post-transcriptionally targeting oncogenes such as *BCL2*, *CDK6*, *MYC*, *SIRT1* and *MET*, thereby causing their suppression. It is frequently found to be downregulated in many cancers including renal, hepatocellular carcinoma, melanoma, and NSCLC [25,98]. Its mimic, MRX34, was developed to restore *miR-34a* and was the first microRNA to have entered human clinical trials for advanced solid tumors. To enhance stability and delivery to the target cells, *miR-34a* was developed as a liposomal nanoparticle with the aim of assessing its antitumor potential, pharmacokinetics, dose limiting toxicities, safety and maximum tolerated dose in the human population (study size–85 patients with advanced solid tumors refractory to standard treatment). The results provided confirmation of the therapeutic potential and clinical feasibility of microRNAs in the treatment of cancers. MRX34 was successfully delivered to the targeted tumor cells and showed antitumor activities in humans with solid tumors with reduced tumor burden in some patients [25,98].

Treatment-attributed serious adverse effects tended to occur after completion of daily MRX34 infusions and included hypoxia, cytokine release syndrome, and hepatic failure, suggestive of immune-mediated toxicity. Following the first incidence of hepatitis, liver function eligibility criteria and monitoring became more stringent. Patients with liver cancer were given allopurinol or its equivalent before the first dose of MRX34 and thereafter to prevent potential tumor lysis syndrome. In summary, the MRX34 trial provided proof of principle that these mimics can be successfully transfected to the targeted tissue and exert biological effects in humans; however, unacceptable toxicities proved to be an a significant concern and resulted in several deaths [98].

In contrast, anti-miR-155 (Cobomarsen), progressed beyond a Phase 1 trial with better tolerability and biological activity. *MiR-155* is a well-studied oncomiR that targets several tumor suppressor genes such as *TP53*, *SOCS1*, *SMAD5*, *BACH1* and *SHIP1*. Its overexpression results in tumor cell proliferation, reduced apoptosis, oncogenesis, and inflammatory signaling. *MicroRNA-155* gene is commonly found overexpressed in many cancers, notably in breast, lung, leukemia, lymphoma and rectal cancer, to name a few, where it activates multiple different cancer-related pathways. It is typically associated with aggressive cancers with poor prognosis and outcome. Unlike *microRNA-34a* mimic, Cobomarsen, an antagomir showed more promising clinical outcomes with less toxicity and better tolerability. It was extensively studied in diffuse large B cell lymphoma and cutaneous T-cell lymphoma involving in vitro and in vivo preclinical trials. In fact, Seto et al. [58] showed that Cobomarsen suppressed several different pathways including that of JAK/STAT and MAPK/ERK thereby reducing proliferation and activating apoptosis in CTCL.

Further validation of Cobomarsen was provided by Anastasiadou et al. [51], who showed reduced proliferation, suppressed tumor growth, and increased apoptosis in in vitro and in vivo. Anastasiadou and colleagues also demonstrated greater tolerability of the drug as reported by a patient who received five doses for treatment of their aggressive ABC-DLBCL. Cobomarsen successfully advanced through Phase I and early Phase II human clinical trials with favorable safety profiles and tolerability with reported side effects described as mild. The results of reduced *miR-155* activity and suppression of oncogenic pathways confirmed that Cobomarsen was an effective oncogenic inhibitor. Despite promising results in the human clinical trial, cobomarsen studies ended in early phase II trial with finances being cited as the reason [51,58], not for toxicity or biological reasons as with MRX34 or for lack of efficacy as with anti-microRNA-21 (Table 1).

Exploring the clinical and therapeutic uses of microRNA in oncology has provided data supporting continued research and development of microRNA-based cancer therapeutics [101]. However, the clinical results from studies of MRX34, Cobomarsen and a few others (Table 2 and Table 3) show a plethora of hurdles encountered in the use of microRNA-targeted cancer treatment. Biological toxicities, efficacy and financial challenges constitute a few of these hurdles. While a number of studies demonstrated strong support for the therapeutic use of microRNAs in cancer, many remain in the preclinical/in vitro phase.

## 4. Modification Strategies for Delivering MicroRNA

### 4.1. Delivery Vehicles

Experimental studies delivered microRNA mimics to targeted cells using methods such as viral vectors, liposomes, plasmids, and polymeric carriers (jetPEI) as well as no carriers. Viral vectors demonstrated efficient transfection, but immunogenicity remained one of the biggest drawbacks [110,111]. Following concerns about the side effects and transient viral gene expression, the liposomal delivery method emerged as a promising alternative offering the advantage of protecting the microRNA mimic from enzymatic degradation, a challenge which was revealed by the delivery of naked microRNAs [25,112]. Early microRNA therapeutic studies using lipofection in vitro showed that, although this method is highly efficient for delivering microRNAs, generalized dose-related cytotoxicity of the lipofection reagents was a significant issue [113]. Specifically, the use of the polyethylenimine (PEI)-based microRNA delivery system has been restricted by its cytotoxic potential, non-specific tissue distribution, and limited in vivo studies. A major turning point for the clinical use of microRNA mimics later demonstrated by Beg et al. [25] was that liposomal MRX34 not only protected the molecule from degradation and subsequent uptake/targeted delivery but also enhanced the half-life in vivo. Unfortunately, as previously noted, it caused severe immune activation and the expected off target biodistribution. One alternative strategy to nanoparticle delivery was provided for *microRNA-145* with an updated adenoviral delivery system (Table 2), which was successful in vivo [46].

These early studies, while demonstrating the feasibility of microRNA therapeutics and their successful target cell delivery, also highlighted drawbacks affecting their clinical translation. This has prompted a focus on clinically optimized RNA platforms that can overcome the issues that have previously limited the use of microRNA therapeutics. Future directions for microRNA delivery include anatomically strategized delivery, the use of targeted nanoparticles, extracellular vesicle-based approaches and antibody-mediated delivery [114,115,116,117,118].

### 4.2. Chemical Modifications of MicroRNA

2′-*O*-methyl modification of microRNA mimics involves the substitution of a hydrogen atom on the RNA ribose with a methyl (CH3) group that sterically changes the molecular conformation, thereby reducing its accessibility to nucleases, protecting it against degradation and ultimately creating longer therapeutic life [119]. Liang et al. [120] showed enhanced stability of *microRNA-21* and improved efficacy when the 2′ hydroxyl group of the microRNA was methylated.

Phosphorothioate, another modification strategy adopted by researchers such as Iwamoto et al. [121], showed that replacing one non-bridging oxygen atom with sulfur in antisense oligonucleotides increases its stability and plasma half-life as well as improves tissue distribution and potency. This modification is commonly used on microRNA mimics. Locked nucleic acid (LNA) modification constrains the ribose ring in a fixed conformation, increasing binding affinity for the complementary RNA target and resistance to degradation. In vitro, an LNA-phosphorothioate microRNA-15a-5p inhibitor reduced microRNA-15a-5p levels in primary human cells without a lipid-based carrier [122].

An in vivo nonhuman primate study found that miravirsen, an LNA-modified anti-microRNA-122, effectively inhibited *microRNA-122* and was generally well tolerated, although transient adverse effects occurred primarily at the highest dose [123]. Miravirsen was developed for chronic hepatitis C virus (HCV) infection, which can lead to cirrhosis and hepatocellular carcinoma.

A Phase 2a trial for miravirsen reported prolonged, dose-dependent reductions in HCV RNA [124]. However, the emergence of direct-acting oral antivirals (DAAs) with cure rates exceeding 95% reduced the clinical need for an injectable therapy, such as miravirsen [125]. Nevertheless, miravirsen provided important proof of concept that an LNA-modified antagomir could achieve therapeutic effects in humans.

Another important modification of microRNA mimics, specifically for cancer treatment, involves substitution of the uracils with 5-fluorouracil (5-FU). This modification has successfully shown passage of modified microRNAs across cell membranes without a nanoparticle or transfection agent [103] (Table 3). This modification may represent a major breakthrough in the use of microRNA mimics for therapeutic use in cancer. Yuen et al. [103] demonstrated that 12 different 5-FU-modified microRNA mimics were able to enter cells without a delivery vehicle, and did so with increased potency (low nanomolar range) in inhibiting cancer cell proliferation [103]. In addition 5-FU toxicity can be predicted pharmacogenomically.

Preclinical investigation of microRNA-129-5p-based therapeutics in Non Small Cell Lung Cancer (NSCLC), led to the development of its modification with 5-fluorouracil, i.e., 5-FU-microRNA-129 [108]. The use of 5-FU-microRNA-129 improved survival with minimal systemic toxicity in in vivo studies using mouse xenograft models. This modified microRNA mimic has not yet transitioned to human clinical trials. Nevertheless, the results are promising and support its potential use for microRNA-based therapeutics.

## 5. What Success in MicroRNA for Cancer Therapeutics Should Resemble

Modification of multiple microRNAs including *let-7-a*, *microRNA-15a*, *-34*, *-129*, *-145*, *-200* (family) and *-155* with subsequent use across various cancer types, showed significant clinical potential. The more recent approach of performing multiple modifications rather than a single modification is being done to further reduce the limitations seen in previous investigational studies with microRNA mimics. This is best demonstrated by the FDA-approved Patisiran, which utilizes a combined chemically modified siRNA and lipid nanoparticle delivery system [126]. Patisiran is a small interfering RNA (siRNA) designed to knock down one specific transcript in a single organ (the liver) for a non-cancerous disease. While not a microRNA, Patisiran represents a related interfering RNA (iRNA) strategy, in that it targets the transthyretin (*TTR*) mRNA in hepatocytes, degrading it and subsequently decreasing the progression of the neurologic disease. The incorporation of 2′-*O*-methylation and 2′-*O*-fluoro modifications in the Patisiran studies (APOLLO clinical trial) resulted in improved pharmacokinetics, resistance, immune tolerance and stability, further validating the potential therapeutic use of iRNA. Patisiran’s approval by the FDA in 2018 represents a huge milestone for the future use of inhibitory RNAs and antagomirs in clinical therapeutics.

While siRNAs usually regulate a specific target transcript, microRNAs such as *microRNA-145* sometimes regulate multiple genes and biological pathways simultaneously. This multi-target functionality (related to more than one cancer characteristic such as failure to undergo cell death and epithelial to mesenchymal transition) may represent an advantage for complex diseases such as cancer, while requiring careful therapeutic optimization.

Patisiran overcame many of the challenges encountered in previous studies, thereby creating a potential framework for future iRNA-based cancer therapy. It is important to highlight that Patisiran achieves liver delivery after intravenous infusion and therefore this siRNA may provide a pathway for success for additional iRNA treatments for hepatic diseases and hepatic cancer. With this example in mind, success of microRNA therapeutics would require one that allows safe and effective delivery with a defined target, minimal off-target effects, low immunogenicity and toxicity and measurable endpoints involving reduced tumor burden and slower cancer progression [127].

## 6. Conclusions

The first obstacle to the use of microRNAs was structural. Historically the proprietary technologies used to stabilize microRNAs for therapeutic and laboratory use have been developed over the last two decades to prevent exonucleolytic damage and stabilization of the phosphodiester backbone. These modifications successfully addressed stability in in vitro settings and in animal models. The issue of delivering microRNA across cell membranes was addressed using nanoparticle transfection delivery systems. In vivo complications have arisen most obviously from the transfection delivery systems, as evidenced by clinical trials. Important outcomes include the studies on MRX34 and cobomarsen. MRX34 demonstrated the feasibility of microRNA therapy but revealed the importance of controlling immune toxicity. Cobomarsen provided useful information regarding the potential of antagomir approaches.

Although siRNA approaches like Patisiran demonstrate specificity to one mRNA, the multifactorial aspect of cancer may call for a microRNA strategy wherein multiple destructive pathways can be targeted simultaneously. The problem of delivering microRNA mimics across cell membranes may be solved by modifications such as the introduction of 5-FU, a cancer drug, for uracil in microRNA molecules. Other emerging technologies for improved clinical translation of microRNA mimics as therapeutics include ligand-conjugated RNA delivery, targeted nanoparticles, extracellular vesicle-based approaches and antibody-mediated delivery.

## Figures and Tables

**Table 1:** Dysregulation of selected microRNAs in cancer with confirmed mRNA targets.

MicroRNA	Cancer Type	Upregulated (OncomiR)	Downregulated (Suppressor)	Targeted RNA	References
*Let-7* family	Breast, lung, ovarian, colorectal, pancreatic, prostate		X	RAS (*K-RAS*, *H-RAS*), *HMGA2*, *Lin-28*	[9,10,11,12,13,14,15,16]
*10b*	Breast	X		*HOXD10*	[17,18]
*21*	Colorectal, multiple myeloma, hepatocellular	X		*PDCD4*, *PTEN*	[19,20,21,22]
*31*	Nasopharyngeal carcinoma, breast		X	*FIH1*, *MCM2*, *SATB2*,	[23,24]
*34a*	Breast, colon, head and neck, thyroid, prostate, pancreatic. Prognostic in NSCLC		X	*E2F*, *SIRT1*	[25,26,27,28,29,30,31,32]
*129*	Lung, colorectal gastric (methylation sensitive miR129)		X	*YWHAB*, *SOX4*, *MCRS1*, *BCL2*	[33,34,35,36,37]
*143*	B cell, colorectal, prostate, renal, breast		X	Hexokinase 2, ERK5, toll like receptor, *HK2*, *ABL2*, *ERBB3*	[38,39,40,41,42,43]
*145*	B cell, gastric, colon, breast adenocarcinoma, liver		X	*MAPK1*, *Fascin1*, *MUC1*, *CDCA3*, *ARF6*, *ERBB3*	[38,43,44,45,46,47,48,49,50]
*155*	Breast, lymphoma, leukemia, lung, pancreatic, thyroid	X		*SHIP1*/*INPP5D*, *SOCS1*, *SOCS6*, *PTEN*, *APC*	[51,52,53,54,55,56,57,58,59,60]
*200*	Breast, ovarian, lung, pancreatic, endometrial, gastric; major regulator of EMT and metastasis		X	*ZEB1*, *SIP1*/*ZEB2*, *VEGFR1*/*Flt1*, *Sec23a*	[61,62,63,64,65,66,67]
*205*	Breast, prostate, lung/NSCLC, head and neck/nasopharyngeal, bladder	X	X	*HER3*/*ERBB3*, *FGF2*, *VEGFA*, *AR*, *PTEN*, *PHLPP2*, *ZEB1*/*SIP1*/*ZEB2*	[63,68,69,70,71,72,73,74,75,76,77]
*221*	Breast cancer, glioblastoma, gastric	X		*PTEN*, *CDKN1B*, *ESR1*, *DNMT3B*, *NOTCH3*, *TRPS1*, *ANXA3*	[78,79,80,81,82,83,84,85,86]
*222*	Breast, thyroid, liver, glioblastoma, prostate, gastric	X		*CDKN1B*/*p27Kip1*, *PTEN*, *NOTCH3*, *ANXA3*	[80,81,82,86,87,88,89,90]

**Table 2:** Summary of in vitro and in vivo use of microRNA mimics or antagomirs with outcomes.

MicroRNA	Studies In Vitro	Studies In Vivo	Cancer Type	Clinical Trial Phase	Outcome
*10b*	Breast cancer	Mouse models Orthotopic metastatic breast cancer mouse models showed prevention or arrest of lymph node metastasis following anti-miR-10b treatment [18]	Metastatic breast cancer	None	Preclinical success for metastasis inhibition [18]
*21*	Multiple myeloma cell lines [20]	Mouse models	Multiple myeloma and cholangiocarcinoma [20,94]	Lademirsen (anti-microRNA-21): in Alport syndrome Reached: Phase II [95]	Terminated for lack of efficacy [95]
*31*	Breast, nasopharyngeal cancer cell lines [23,96]	Mouse metastasis models	Breast cancer [96]	none	Preclinical success only
*34* *34a*	Breast cancer cell [97]	C. elegans with loss-of-function mutations in the *mir-34* gene [97] Human [95]	Solid tumors (hepatocellular carcinoma, melanoma, NSCLC, renal cancer)	Phase I [95]	Trial terminated due to severe immune-mediated toxicity; 4 treatment-related deaths [25,98]
*129*	Colorectal cancer, gastric cancer, leukemia cell lines [95]	Mouse xenografts	Colorectal cancer [33]	none	Tumor suppressor effects preclinically
*143*	Colon cancer cell lines [40], Prostate cancer [39]	Mouse xenografts	Prostate cancer [39], colorectal cancer [41], renal cancer [42]	Observational	Reduced tumor volume
*145*	Breast cancer [46], colon adenocarcinoma cell lines [49], liver [99]	Mouse xenografts adenoviral, delivery of miR (ad-miR), rectal cancer patient-derived xenografts (PDXs)	Breast cancer [46]	none	Preclinical efficacy
*155*	T and B cell lymphoma, leukemia cell lines [52,58]	Mouse xenografts	T and B cell lymphoma, leukemia [52]	Cobomarsen (MRG-106) used in CTCL, T-cell leukemia and lymphoma. Phase I and Phase II [51,58]	Partial responses, financial-program issues that led to premature termination: phase I trial was completed, two of the phase II studies were terminated [51,58]
*200*	Breast cancer cell lines [63]	Mouse models	Breast cancer [64]	none	Successful preclinically
*205*	Breast cancer cell lines [77], prostate cancer [70]	Tumor tissue, cancer cell lines in mouse xenografts [77]	Breast cancer [77]	none	Successful preclinically

**Table 3:** Modifications of microRNAs.

MicroRNA	Modification	Outcome	Cancer Type
*Let7a* [102,103]	5FU	Increased in vitro efficacy to inhibit cancer cell proliferation compared to the unmodified microRNA.	Gastric, lung Biodegradable Nanoassemblies for *KRAS* mutated cancer [104]
*MiR15* [105,106,107]	5FU	Increased in vitro and in vivo (mouse colon cancer model) efficacy to inhibit cancer cell proliferation compared to the unmodified microRNA.	Colon, pancreas, breast
*MiR34* [25]	Liposome formulation (microRNA mimic)	Increased stability of nucleic acid and decreased nucleic acid enzymatic degradation by serum nuclease in human (clinical trial phase I).	Solid tumors
*MiR129* [107,108]	5FU	Increased in vitro efficacy to inhibit cancer cell proliferation compared to the unmodified microRNA.	Colorectal, pancreatic, breast
*MiR145* [50,109]	5FU	Increased in vitro efficacy to inhibit cancer cell proliferation compared to the unmodified microRNA.	Gastric, lung
*MiR155* [51,52,58]	Locked nucleic acid-based (Cobomarsen: antagomir of *miR-155*)	Cobomarsen decreased cancer cell proliferation and induced apoptosis, reduced tumor volume and derepressed direct *miR-155* target genes in human (clinical trial phase II).	Lymphoma, leukemia
*MiR200* [65,66,67]	5FU	Increased in vitro efficacy to inhibit cancer cell proliferation compared to the unmodified microRNA.	Breast, gastric, lung, pancreatic

## Data Availability

No new data were created or analyzed in this study. Data sharing is not applicable to this article.
